# Labdane and Abietane Diterpenoids from *Juniperus oblonga* and Their Cytotoxic Activity

**DOI:** 10.3390/molecules24081561

**Published:** 2019-04-19

**Authors:** Yilin Qiao, Manana Khutsishvili, Valida Alizade, Daniel Atha, Robert P. Borris

**Affiliations:** 1 School of Pharmaceutical Science and Technology, Health Sciences Platform, Tianjin University, Tianjin 30072, China; qiaoyilin_128@tju.edu.cn; 2 National Herbarium of Georgia, Ilia State University, Tbilisi 100995, Georgia; mananakhuts@yahoo.com; 3 Institute of Botany, Azerbaijan National Academy of Sciences, Baku AZ1102, Azerbaijan; vm_alizade@yahoo.com; 4 New York Botanical Garden, Bronx, NY 10041, USA; datha@nybg.org

**Keywords:** Cupressaceae, *Juniperus oblonga*, labdane diterpenoid, CD exciton chirality method, cytotoxic activity

## Abstract

A phytochemical investigation of the whole plant of *Juniperus oblonga* led to the isolation of one previously undescribed labdane diterpenoid, (4*R*,5*S*,9*S*,10*R*)-13-des-ethyl-13-oxolabda-8(17),11*E*-dien-19-oic acid (**1**), together with nine known diterpenoids (**2**–**3**, **6**–**12**), two lignans (**4**, **5**), and a coumarin (**13**). The structures of all the compounds were elucidated on the basis of spectrometric data, primarily one-dimensional (1D)- and two-dimensional (2D)-NMR and mass spectrometry. Electronic circular dichroism (ECD) calculations determined the absolute configuration of **1**. In addition, the isolated compounds were evaluated for their cytotoxic activity against three human tumor cell lines (HepG2, MCF-7, and HeLa). 6,12-Dihydroxyabieta-5,8,11,13-tetraen-7-one (**6**) showed moderate cytotoxicity against all three cell lines with IC_50_ values ranging from 24.41 μM to 58.39 μM and trilobinone (**10**) showed weaker activity with IC_50_ values ranging from 56.93 μM to 79.98 μM. None of the isolated diterpenoids have been previously reported from *Juniperus* *oblonga*, and five compounds are here reported from the genus *Juniperus* for the first time.

## 1. Introduction

*Juniperus oblonga* M. Bieb. belongs to the family Cupressaceae (Cypress family). The genus *Juniperus* is one of the largest conifer genera and it is widely distributed in the temperate regions of the Northern Hemisphere [1]. *Juniperus* is a well-known source of folk medicines in several parts of the world [2]. For traditional medicine, some species represent drugs with several properties, such as antitussive and haemostatic activities [3], antifertility effect [4], and antitumor activity [5]. The berries also have antimicrobial activity and anti-hypercholesterolemic activity [6,7]. The *Juniperus* species are used as an insect repellent and in the treatment of fever and dysuria in Bhutan [8]. *Juniperus oblonga* belongs to the subgenus *Oxycedrus* of the genus *Juniperus.* Its ripe berries have been found to exert diuretic and antiscorbutic effects [9], and the essential oils that were obtained from the fruits and branchlets of this plant possess antioxidant and anti-glycation properties [10].

Diterpenoids that were extracted from *Juniperus* species are mainly based on abietane and labdane skeleta. Many abietane diterpenoids function as ecophysiological mediators, especially defense chemicals, and they exhibit broad biological activities, including anticancer [11], antimicrobial [12], anti-tumor-promoting activity [13], and antiulcerogenic effects [14]. The labdane diterpenes have also been shown to possess cardiovascular effects [15], anti-fungal activity [16], and anti-inflammatory and cytotoxic effects [17].

Our continuing phytochemical investigation on *Juniperus oblonga* has led to the discovery of one undescribed labdane diterpene, nine known diterpenes, two known ligans, and a coumarin. Herein we report the isolation and structural elucidation of the undescribed diterpenoid by extensive spectroscopic techniques and chemical means. The CD exciton chirality method and calculated ECD spectra determined the absolute configuration of the new compound. X-ray diffraction analysis determined the crystal structures of compound **3** and **6**. This is the initial report of the crystal structure of **6**. The cytotoxicity of the isolated compounds was evaluated against three human tumor cell lines—HepG2, MCF-7, and HeLa.

## 2. Results and Discussion

**Figure 1:**
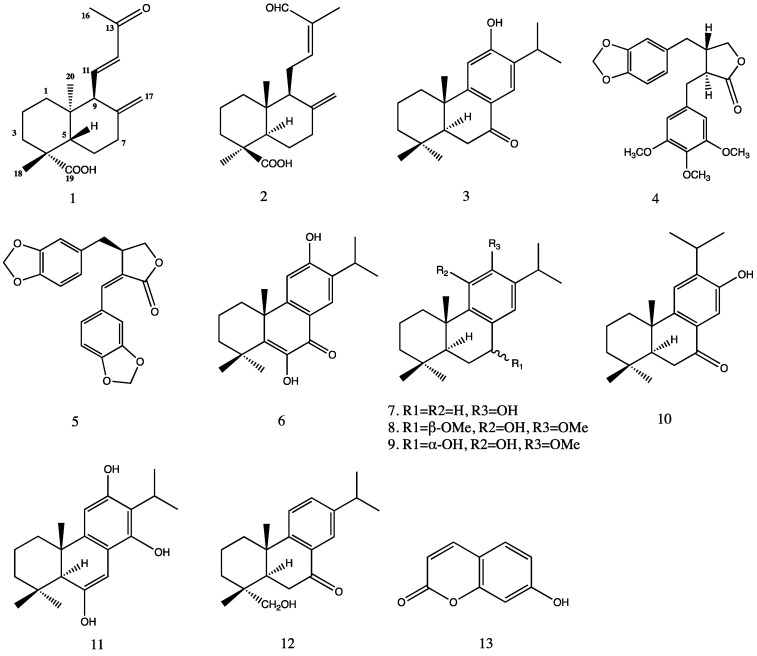
Structures of compounds **1**–**13**.

Compound **1** was isolated as a white amorphous powder from the dichloromethane extract of the roots, stems, leaves, and fruits of *Juniperus oblonga*. The UV spectrum showed absorption maxima at 246 and 231 nm. Its optical rotation was determined as [α]^20^_D_ = +8.0 (c 0.2 DMSO). The high-resolution mass spectrum (Figure S1, Supplementary Material) of **1** displayed a deprotonated molecular ion [M − H]^−^ at *m*/*z* 289.1809 (calcd. 289.1804), which corresponded to formula C_18_H_26_O_3_, accounting for six degrees of unsaturation. The ^1^H-NMR, ^13^C-NMR, and DEPT135 data of **1** (Table 1) suggested a labdane diterpene skeleton (Figure 1). In the ^13^C-NMR spectrum, two carbonyl carbons, C-13 (δ_C_ 200.9), C-19 (δ_C_ 181.2), are observed, along with four olefinic carbon signals at C-8 (δ_C_ 149.9), C-11 (δ_C_ 148.7), C-12 (δ_C_ 134.6), and C-17 (δ_C_ 108.9). The chemical shifts of C-17 and C-8 are typical of an exocyclic methylene group in a labdane skeleton. Three methyl singlets at δ_H_ 1.21, 2.27, and 0.83 ppm showed HSQC correlations with the carbons at δ_C_ 29.4, 27.1, and 14.2 ppm.

**Figure 2:**
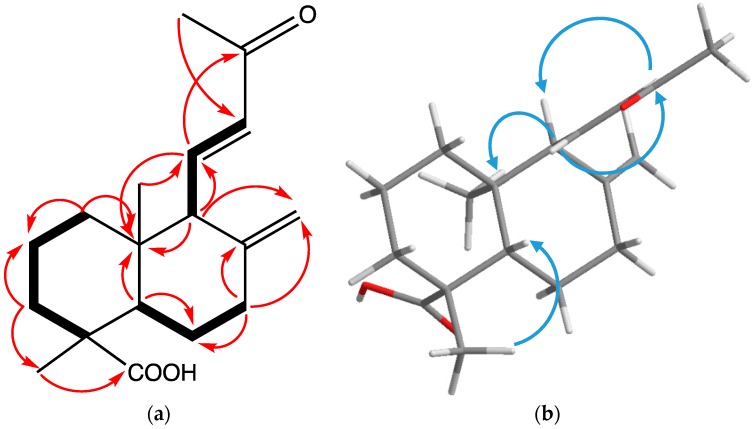
Some of the key correlations observed for compound **1**. (**a**) ^1^H-^1^H COSY (bold) and key HMBC (red single headed) correlations of compound **1**. (**b**) NOESY (blue single headed) correlations of compound **1**.

From the COSY spectrum (Figure 2), correlations between H_2_-1 (δ_H_ 1.42, 1.14), H_2_-2 (δ_H_ 1.88, 1.43), and H_2_-3 (δ_H_ 1.08, 2.14) suggested a typical A-ring skeleton of a diterpene without further substitution. HMBC correlations were observed between H_2_-3 and the methyl group, C-18 (δ_C_ 29.4), and a carboxylic acid, C-19, (Figure 2). Additionally, C-19 showed HMBC correlations, with H-5 (δ_H_ 1.44) and H-18 (δ_H_ 1.21) establishing the location of both the carboxylic acid and methyl group at C-4. COSY correlations of H_2_-6 (δ_H_ 2.00, 1.93) to H-5 and H_2_-7 (δ_H_ 2.45, 2.06) were observed, as well as HMBC correlations between H_2_-7 and both C-8 and C-17, establishing the position of the exocyclic double bond. Observation of HMBC correlations from H-9 (δ_H_ 2.57) to C-5 (δ_C_ 56.4) and C-17 further supported this assignment. COSY correlations from H-11 (δ_H_ 6.95) to H-12 (δ_H_ 6.08) and H-9 established that the second double bond was attached at C-9. The ketone carbonyl carbon, C-13, displayed HMBC correlations, with H-11, H-12, and a methyl group, H_3_-16, while H-12 showed correlations with C-13 and C-16, establishing the structure of the side chain. Correlations in the NOESY spectrum from H-11 to H_3_-20 (-CH_3_) and H_3_-16 (-CH_3_) (Figure 2) showed the spatial proximity of these groups, while correlations between H-12 and H-9, H-5, and H-18 supported a chair conformation for both rings. Based on these observations, the structure of **1** was determined to be 13-des-ethyl-13-oxolabda-8(17),11*E*-dien-19-oic acid.

**Figure 3:**
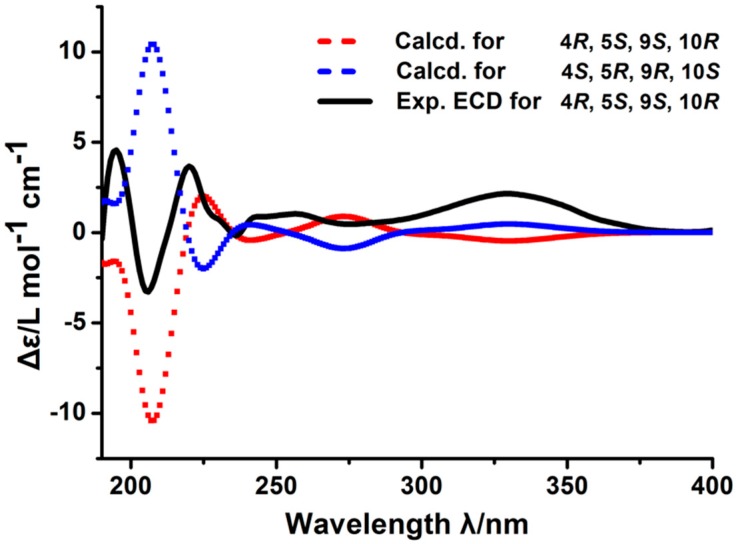
Electronic circular dichroism (ECD) spectrum of compound **1** (The black line is the experimental ECD spectrum, red and blue dashed lines are calculated ECD spectra.).

Applying the CD exciton chirality method determined the absolute configuration of compound **1**. The experimental ECD spectrum of **1** showed Cotton effects (CEs) at 200–250 nm, including a positive CE at 220 nm, which is indicative of a butene moiety and a negative CE at 205 nm due to a n → π transition. The calculated ECD spectrum for the 4*R*, 5*S*, 9*S*, and 10*R* configuration matched the experimental data of compound **1**. Therefore, the absolute configuration of **1** is (4*R*,5*S*,9*S*,10*R*)-13-des-ethyl-13-oxolabda-8(17),11*E*-dien-19-oic acid. (Figure 3)

**Figure 4:**
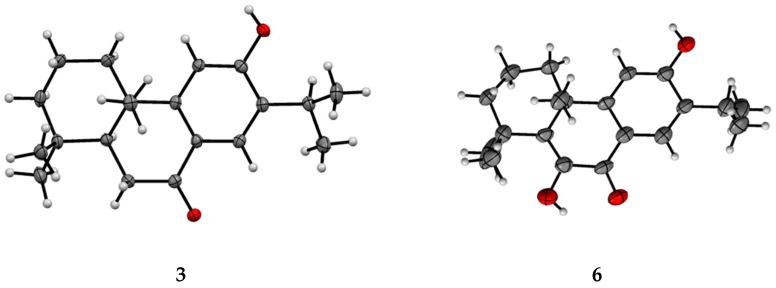
Crystal structure representations of compound **3** and **6**. The structure of **6** was deposited with the Cambridge Crystallographic Data Centre (CCDC) code 1900758.

Single crystal X-ray diffraction studies allowed for the elucidation of the structures of **3** and **6** as sugiol (**3**) [18], and 6,12-dihydroxyabieta-5,8,11,13-tetraen-7-one (**6**) [19] (Figure 4). While the crystal structure of sugiol has been previously reported [18], this is the first report of the crystal structure of **6**, which has a CCDC (Cambridge Crystallographic Data Centre) code: 1900758. Supplementary Information (Tables S1–S7) presents the X-ray diffraction data, parameters, bond lengths, and bond angles.

The remaining compounds were identified by comparison of their spectroscopic data with those that were reported in the literature. The other seven diterpenoids were identified as 15-nor-14-oxolabda-8(17),12*Z*-dien-19-oic acid (**2**) [20], ferruginol (**7**) [21], 7α-methoxydeoxocryptojaponol (**8**) [22], 7β-hydroxydeoxocryptojaponol (**9**) [23], sempervirol (**10**) [24], trilobinol (**11**) [25], and 7-oxodehydroabietinol (**12**) [26]. Two lignans were identified as (−)-yatein (**4**) [27] and helioxanthin (**5**) [28]. One coumarin was determined as umbelliferone (**13**) [29]. To date, none of these diterpenoids have been reported from *Juniperus oblonga*. (4*R*,5*S*,9*S*,10*R*)-13-des-ethyl-13-oxolabda-8(17),11*E*-dien-19-oic acid (**1**), 15-nor-14-oxolabda-8(17),12*Z*-dien-19-oic acid (**2**), sempervirol (**10**), trilobinol (**11**), and 7-oxodehydroabietinol (**12**) are first reported here from the genus *Juniperus*.

When isolated quantities permitted, the isolated compounds were evaluated for cytotoxicity against three cancer cell lines, including a human hepatocellular carcinoma cell line (HepG2),a human breast cancer cell line (MCF-7) and a human cervical carcinoma cancer cell line (HeLa), as well as a normal human liver cell line (LO2), at an initial concentration of 4 mg/mL (Table 2) using a standard MTT assay [30]. The IC_50_ values were determined for compounds that showed greater than 50% inhibition at this concentration. Compound **6** exhibited moderate cytotoxicity against all three cell lines, with IC_50_ values ranging from 24.41 to 58.39 μM, while **10** showed weaker activity, with IC_50_ values that ranged from 56.93 to 79.98 μM (Table 3). The remaining compounds did not exhibit cytotoxicity. The absence of cytotoxicity against the normal human liver cell line (LO2) may have some importance. While none of the compounds that were isolated in this study have the potential for development into clinically useful anticancer agents, other labdane and abietane diterpenoids have shown antibacterial, antifungal, and anti-inflammatory activities [17,31,32,33]. Members of these diterpene families not being cytotoxic to normal human cells may enhance their potential for development for these other bioactivities.

## 3. Materials and Methods

### 3.1. General Experimental Procedures

ECD measurements were determined on a BioLogic SAS MOS-500 spectropolarimeter (Bio-Logic SAS, Claix, France). Analytical and semipreparative HPLC separations were performed using an Agilent 1260VL instrument (G1311C pump, G1329B autosampler, G1316A thermostatted column compartment and G1315D photodiode array detector, Agilent Technologies, Santa Clara, CA, USA). Analytical HPLC separations were carried out using a SunFire C18 column (3.5 μm particles, 2.1 × 150 mm, Waters Corp., Milford, MA, USA). Semi-preparative and preparative HPLC separations were performed on a Hypersil Gold C18 column (5 μm particles, 10 × 250 mm, Thermo Scientific, Waltham, MA, USA) and a Hypersil Gold C18 column (5 μm particles, 21.2 × 250 mm, Thermo Scientific). The UV spectra were recorded using a Hitachi U-3900 spectrophotometer (Hitachi Limited, Tokyo, Japan). The samples were routinely evaporated while using a Thermo Scientific SC210A SpeedVac concentrator. NMR spectra were recorded on Bruker Avance III 600MHz and 400MHz spectrometers (Bruker-Biospin Corp., Billerica, MA, USA). Residual solvent resonances were used as the internal reference, and chemical shifts are reported in δ (parts per million). Mass spectra were determined on an Agilent Technologies 6230 TOF LC-MS or an Agilent Technologies 6420 triple quadrupole LC-MS using electrospray ionization in the positive and negative modes. Column chromatography was performed on silica gel (60 Å, 40–63 μm) that was purchased from Sorbent Technologies (Norcross, GA, USA). All of the solvents used for isolation were of HPLC grade (Concord Technology, Tianjin, China). Dulbecco’s modified Eagle’s medium (DMEM), Roswell Park Memorial Institute 1640 (RPMI-1640), Phosphate buffered saline (PBS), and fetal bovine serum (FBS) were purchased from Gibco Laboratories (Gaithersburg, MD, USA). Penicillin-Streptomycin solution (100X) was purchased from Beijing Solarbio Science and Technology (Beijing, China). 3-(4,5-Dimethylthiazol-2-yl)-2,5-diphenyl tetrazolium bromide (MTT) was purchased from Biotopped Technology (Beijing, China).

### 3.2. X-ray Diffraction Analyses

The single crystal X-ray diffraction data were collected on a ROD, Synergy Custom system, HyPix diffractometer (Rigaku, Japan). The crystal was maintained at 159.99(10) K during data collection. The structures were solved using Olex2 with the ShelXT structure solution program using intrinsic phasing and refined with the ShelXL refinement package while using least squares minimization.

### 3.3. Plant Material

Samples of the roots, stems, leaves, and fruits of *Juniperus oblonga* M. Bieb. (Cupressaceae) were individually collected near Kish Village in the Sheki District of Azerbaijan in September 2006. Herbarium specimens documenting the collection (Kerimov 57) have been deposited in the herbaria of the Institute of Botany, Azerbaijan National Academy of Sciences (BAK) and the New York Botanical Garden (NY).

### 3.4. Extraction and Isolation

Fresh plant samples were freed of extraneous matter, air-dried, and then milled to a coarse powder. A 1 kg portion of each dried sample was extracted with methanol (3 × 4 L). After the removal of solvent, the resulting viscous oil was dispersed in 1 L of methanol: water (9:1) and extracted with *n*-hexane (3 × 1 L). The hexane-depleted hydroalcoholic phase was freed of methanol, dispersed in distilled water (1 L), and then sequentially extracted with dichloromethane and water saturated n-butanol (each 3 × 1 L). The resulting solvent-soluble fractions were each evaporated to dryness in vacuo, while the residual aqueous fraction was freed of solvent and lyophilized. The dichloromethane part (7.46 g) was fractionated by column chromatography on silica gel, eluting with a step-gradient of CH_2_Cl_2_/MeOH (100:0, 99:1, 98:2, 97:3, 95:5, 90:10, 80:20, 50:50, 100:0) to give twenty-seven fractions (Fr. 1–Fr. 27).

Compound **3** was crystallized by the slow evaporation of the CH_2_Cl_2_/MeOH mixture from Fr. 5.

Fr. a (1.1 g), formed by combining fraction 5 (after depletion of **3** by crystallization) with fractions 6–13, was subjected to silica gel column chromatography eluting with a step- gradient of *n*-hexane/ethyl acetate (8:2, 7:3, 6:4, 5:5, 0:10) to yield five fractions (Fr. a-1 to Fr. a-5). Fr. a-3 (290 mg) was separated by preparative HPLC eluting with a linear gradient of ACN/H_2_O (20–100% ACN, 10–50 min) that contained 0.1% formic acid at 10 mL/min to afford four subfractions (Fr. a-3-1 to Fr. a-3-4).

Fr. a-3-2 (78 mg) was purified by preparative HPLC eluting with ACN/H_2_O (40:60) containing 0.1% formic acid at 10 mL/min, affording **2** (1 mg). Further fractionation by semi-preparative HPLC eluting with ACN/H_2_O (45:55) containing 0.1% formic acid at 4 mL/min afforded compound **13** (1.1 mg).

Fr. a-3-3 (100 mg) was purified by preparative HPLC eluting with ACN/H_2_O (35:65) containing 0.1% formic acid at 10 mL/min, to afford compound **1** (2.3 mg), **4** (1.4 mg), and **5** (1.1 mg).

Fractions 2-4 were pooled to form Fr. b (240 mg), which was subjected to silica gel column chromatography, eluting with a step-gradient of *n*-hexane/ethyl acetate (95:5, 92.5:7.5, 90:10, 80:20, 50:50, 0:100) to give eighteen fractions (Fr. b-1 to Fr. b-18).

Fr. b-1 (57 mg) was purified by semi-preparative HPLC eluting with ACN/H_2_O (10:90) containing 0.1% formic acid at 4 mL/min, affording compound **12** (2.3 mg).

Fr. b-3 (35 mg) was purified by semi-preparative HPLC eluting with ACN/H_2_O (65:35) containing 0.1% formic acid at 4 mL/min, affording compound **7** (1.5 mg) and **8** (2 mg).

Fr. b-5 (48 mg) was purified by semi-preparative HPLC eluting with ACN/H_2_O (60:40) containing 0.1% formic acid at 4 mL/min, affording compound **6**, **9** (1.3 mg), and **10** (2.5 mg). Compound **6** (2.1 mg) was crystallized by the slow evaporation of the ACN/H_2_O mixture.

Fr. b-9 (10 mg) was subjected to semi-preparative HPLC eluting with ACN/H_2_O (55:45) containing 0.1% formic acid at 4 mL/min, to afford compound **11** (1 mg).

(4*R*,5*S*,9*S*,10*R*)-13-Des-ethyl-13-oxolabda-8(17),11*E*-dien-19-oic acid (**1**): Colorless amorphous powder; UV (DMSO) λ_max_ (log ε) 246 (3.05) and 231 (2.52) nm; HR-TOF-MS *m*/*z* 289.1809 [M − H]^−^, (calcd. for C_18_H_25_O_3_^−^, *m*/*z* 289.1804). ECD (DMSO) λ (Δε) 205 (−3.14), 220 nm (+3.52). ^1^H-NMR and ^13^C-NMR spectral assignments, see Table 1.

### 3.5. Single Crystal X-ray Diffraction Analysis

#### 3.5.1. Crystallographic Data for **3**

The crystal data for **3** (C_20_H_28_O_2_) is clear light colorless needle crystal, crystal size 0.2 × 0.2 × 0.2 mm^3^, orthorhombic, space group P2_1_2_1_2_1_ (no. 19), *a* = 9.54890(10) Å, *b* = 12.6943(2) Å, *c* = 14.1587(2) Å, *V* = 1716.27(4) Å^3^, Z = 4, *T* = 100.0(3) K, μ (Cu Kα) = 0.565 mm^−1^, *Dcalc* = 1.163 g/cm^3^, 9031 reflections measured (9.356° ≤ 2θ ≤ 148.94°), 3362 unique (R_int_ = 0.0317, R_sigma_ = 0.0323), which were used in all of the calculations. The final *R*_1_ was 0.0331 (I > 2σ(I)) and *wR*_2_ was 0.0867 (all data).

#### 3.5.2. Crystallographic Data for **6**

The crystal data for **6** (C_20_H_26_O_3_) is colorless needle crystal, crystal size 0.15 × 0.15 × 0.2 mm^3^, orthorhombic, space group P2_1_2_1_2_1_ (no. 19), *a* = 10.4261(4) Å, *b* = 14.6852(6) Å, *c* = 23.2228(12) Å, *V* = 3555.6(3) Å^3^, *Z* = 8, *T* = 159.99(10) K, μ (Cu Kα) = 0.614 mm^−1^, *Dcalc* = 1.175 g/cm^3^, 12,897 reflections measured (7.122° ≤ 2θ ≤ 154.618°), 6523 unique (R_int_ = 0.1032, R_sigma_ = 0.1098), which were used in all calculations. The final *R*_1_ was 0.0945 (I > 2σ(I)) and *wR*_2_ was 0.2949 (all data).

CCDC 1900758 contains the supplementary crystallographic data for this paper. These data can be obtained free of charge via http://www.ccdc.cam.ac.uk/conts/retrieving.html (or from the CCDC, 12 Union Road, Cambridge CB2 1EZ, UK; Fax: +44 1223 336033; E-mail: deposit@ccdc.cam.ac.uk).

### 3.6. Cytotoxicity Assay

MTT assay was used to measure the in vitro cytotoxicity of the isolated compounds. Human breast adenocarcinoma cell line (MCF-7), human liver hepatocellular carcinoma cell line (HepG2), and human cervical cancer cell line (HeLa) were cultured in Dulbecco’s modified Eagle’s medium (DMEM) and Roswell Park Memorial Institute medium (RPMI 1640), which was supplemented with 10% fetal bovine serum (FBS) at 37 °C in a humidified atmosphere of 5% CO_2_. The cells were cultured in 96-well plates for 24 h and then treated with test compounds at various concentrations (8–125 μM) for 72 h. After incubation for another 4 h with a 20 µL aliquot of the MTT [3-(4,5-dimethylthiazol-2-yl)-2,5-diphenyl tetrazolium bromide] solution (5 mg/mL in PBS), the medium was discarded, and 150 µL of DMSO was added to dissolve the produced formazan. The absorbance was measured at 490 nm and 570 nm using a microplate reader. Doxorubicin was used as a positive control. Each experiment was carried out in triplicate. The IC_50_ values were calculated using Graphpad Prism 5 software.

### 3.7. Calculations of the CD Spectra

The theoretical calculations of the model molecules were carried out using Gaussian 09. MMFF94 was used to initially perform conformational analysis. The conformers were optimized at the B3LYP/6-31 G (d) level. Room temperature equilibrium populations were calculated according to the Boltzmann distribution law. Table S7 (Supplementary Material) show the optimized conformation geometries, thermodynamic parameters, and populations.

## 4. Conclusions

Thirteen compounds, including one previously undescribed labdane diterpene, nine known diterpenoids, two lignans, and a coumarin were isolated from *Juniperus oblonga*, and their structures were primarily elucidated on the basis of NMR and MS studies. The absolute configuration of the new compound was determined by CD exciton chirality and calculated ECD methods. The crystal structures were determined for two of the diterpenes (**3** and **6**) by single crystal X-ray diffraction analyses. The isolated compounds were tested for cytotoxicity against three cell lines, with **6** showing moderate cytotoxicity against all three cell lines and **10** exhibiting weaker activity.

## Figures and Tables

**Table 1:** NMR spectroscopic data for compound **1** acquired in CD_3_OD.

Position	δ_C_	δ_H_, *J* (Hz)	^1^H-^1^H-COSY	HMBC	NOESY
1	42.2	1.42 m, 1.14 ddd 4.0, 13.0	H-2	C-2, C-10, C-20	
2	20.9	1.88 td 3.0,13.0, 1.43 m	H-1, H-3	C-3, C-5	
3	39.3	2.14m, 1.08 ddd 3.4, 13.0	H-2	C-2, C-4, C-5, C-18, C-19	
4	45.1				
5	56.4	1.44 m	H-6	C-6, C-10, C-19, C-20	H-18
6	26.4	2.00m, 1.93 ddd 3.8, 13.0	H-7	C-5, C-7	
7	38.3	2.45 dt 3, 12, 2.06 ddd 5.0,13.0	H-6	C-5, C-8, C-6, C-17	
8	149.9				
9	61.4	2.57 d 10	H-11	C-5, C-10, C-11, C-12, C-17, C-20	H-12
10	41.0				
11	148.7	6.95 dd 5.0 10.0	H-9, H-12	C-8, C-9, C-10, C-13	H-12, H-14, H-20
12	134.6	6.08 d 5.0	H-11	C-8, C-9, C-13, C-16	H-11
13	200.9				
16	27.1	2.27 s		C-11, C-12, C-13	H-11,
17	108.9	4.80 d 1.5, 4.42 d 1.5		C-7, C-8, C-9	
18	29.4	1.21 s		C-2, C-3, C-4, C-5, C-19	H-5
19	181.2				
20	14.2	0.83 s		C-5, C-9, C-10	H-11

**Table 2:** Cytotoxicity (%I @ 4 mg/mL)) of compounds from *Juniperus oblonga* against various cell lines.

Compound	HepG2	HeLa	MCF-7	LO2
**1**	18.39	26.36	28.45	20.57
**2**	16.06	23.95	21.08	10.61
**3**	35.05	12.29	9.2	26.62
**5**	0.11	0.22	10.09	9.05
**6**	86.57	83.83	83.99	27.76
**8**	7.84	0	1.35	17.07
**9**	24.33	0	10.96	22.21
**10**	48.67	63.4	43.4	19.08
**11**	67.12	22.01	29.88	23.29
**13**	32.71	3.6	17.96	26.59
Doxorubicin ^a^	42.21	49.51	46.46	46.7

^a^: Doxorubicin was tested as a positive control.

**Table 3:** Cytotoxicity of compounds **6** and **10** against three human cancer cell lines.

Compounds	Cytotoxicity (IC_50_: μM) ^a^		
	HepG2	MCF-7	Hela
**6**	48.73 ± 1.31	58.39 ± 2.45	24.41 ± 2.05
**10**	64.94 ± 2.64	79.98 ± 1.20	56.93 ± 2.39
Doxorubicin ^b^	3.18 ± 1.19	3.44 ± 1.59	3.64 ± 1.37

^a^ IC_50_ values are expressed as the mean values of three experiments ± SD; ^b^ Doxorubicin was tested as a positive control.

## Supplementary Materials

The following are available online: NMR, MS data of compound **1**, data of ECD calculation for compound **1**, single crystal X-ray diffraction data and refinement parameters with bond lengths and bond angles of compound **3** and **6**. IC_50_ valves of selected compounds.
