# Supplementary material for: Labdane and Abietane Diterpenoids from Juniperus oblonga and Their Cytotoxic Activity
Source: Molecules. 2019 Apr 19;24(8):1561. doi: 10.3390/molecules24081561 (PMC6514680; doi:10.3390/molecules24081561)
Supplement: Supplementary file 1 [file molecules-24-01561-s001.pdf]

Labdane and abietane diterpenoids from *Juniperus oblonga* and their cytotoxic activity

Yilin Qiao[1], Manana Khutsishvili[2], Valida Alizade[3], Daniel Atha[4], and Robert P. Borris[2,*]

[1] School of Pharmaceutical Science and Technology, Health Sciences Platform, Tianjin University, Tianjin, 30072, CHINA; qiaoyilin_128@tju.edu.cn

[2] National Herbarium of Georgia, Ilia State University, Tbilisi, 100995, GEORGIA; mananakhuts@yahoo.com

[3] Institute of Botany, Azerbaijan National Academy of Sciences, Baku, AZ1102, AZERBAIJAN; vm_alizade@yahoo.com

[4] New York Botanical Garden, Bronx, NY, 10041, USA; datha@nybg.org

* Correspondence: Email: rborris@tju.edu.cn Phone: +86 183 0224 2039

Acquisition Parameter

Method:	MS-MS.m			Acquisition Date:	1/4/2019 2:32:20 PM
File Name:	D:\Data\YSY\20190104\QYL-2_P1-A-3_01_8241.d			Operator:	Shuyang Yang / XZ
Source Type	ESI	Ion Polarity	Negative	Set Nebulizer	0.8 Bar
Focus	Active	Set Capillary	2500 V	Set Dry Heater	200 °C
Scan Begin	50 m/z	Set End Plate Offset	-500 V	Set Dry Gas	4.0 l/min
Scan End	2800 m/z	Set Collision Cell RF	200.0 Vpp	Set Divert Valve	Source

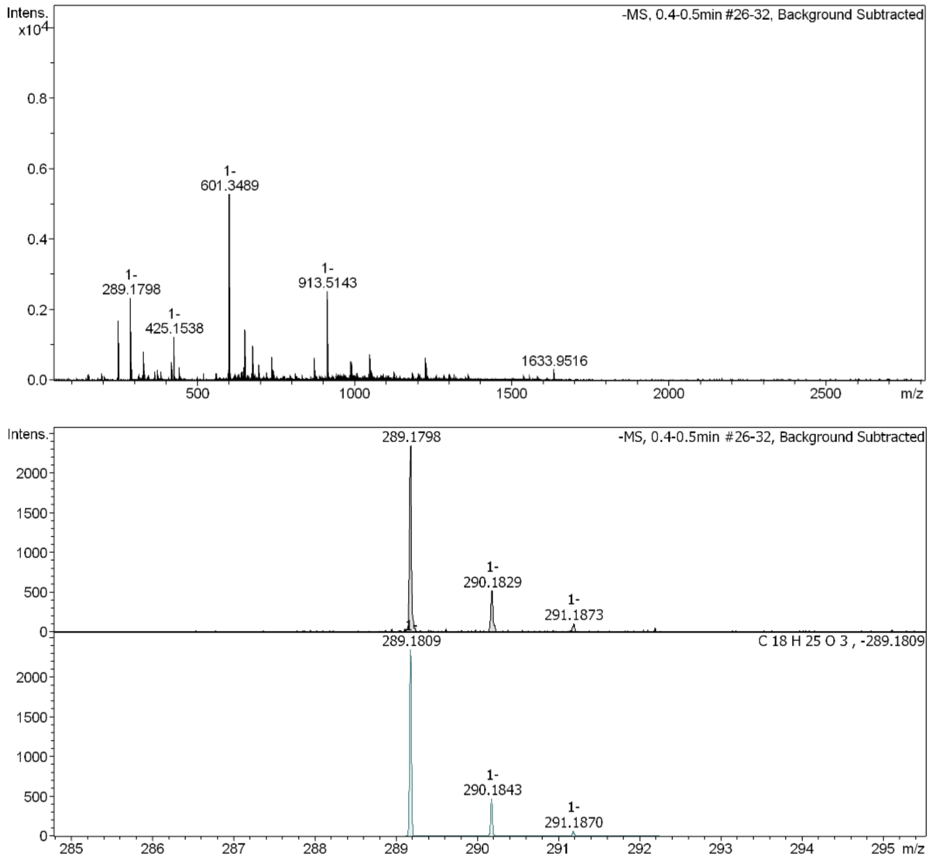

Bruker Daltonics ESI - micrOTOF Q II   MS Lab | IAC - SPST - TJU   Page 1 of 2

Fig. 1S. HR-ESI-MS spectrum of **1**

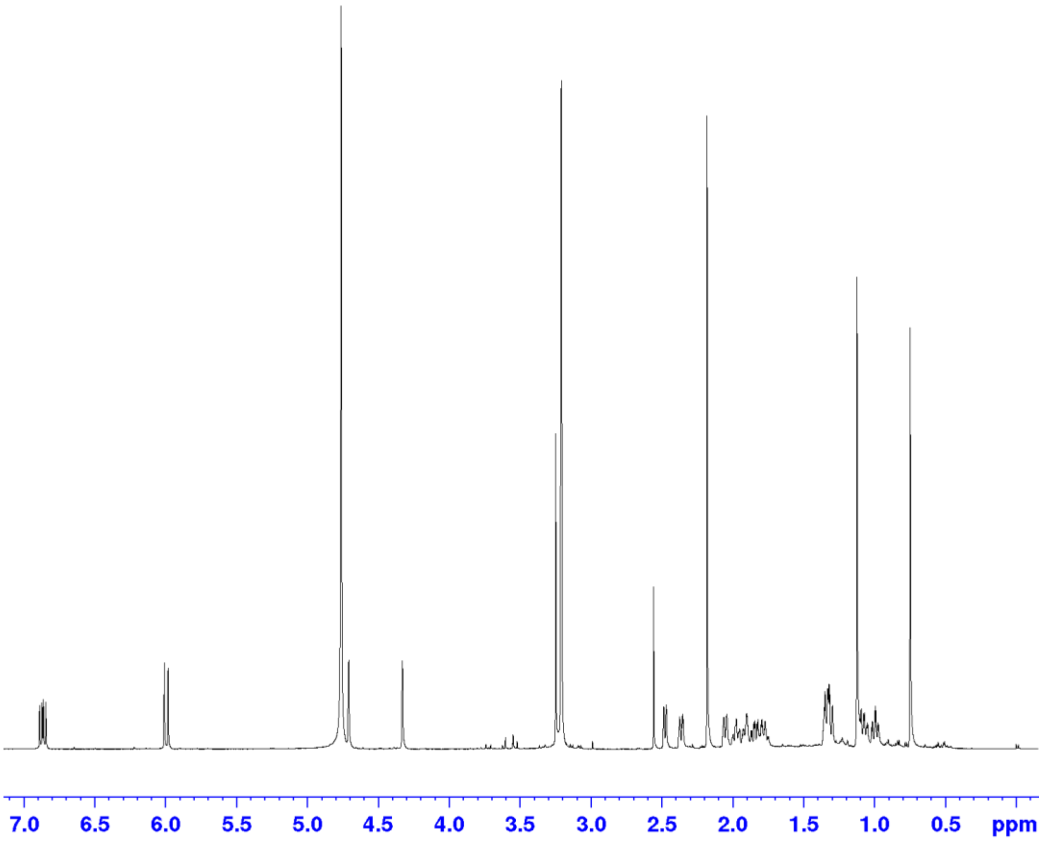

Fig. 2S. ^{1}H-NMR spectrum of **1** (600MHz, CD_3OD)

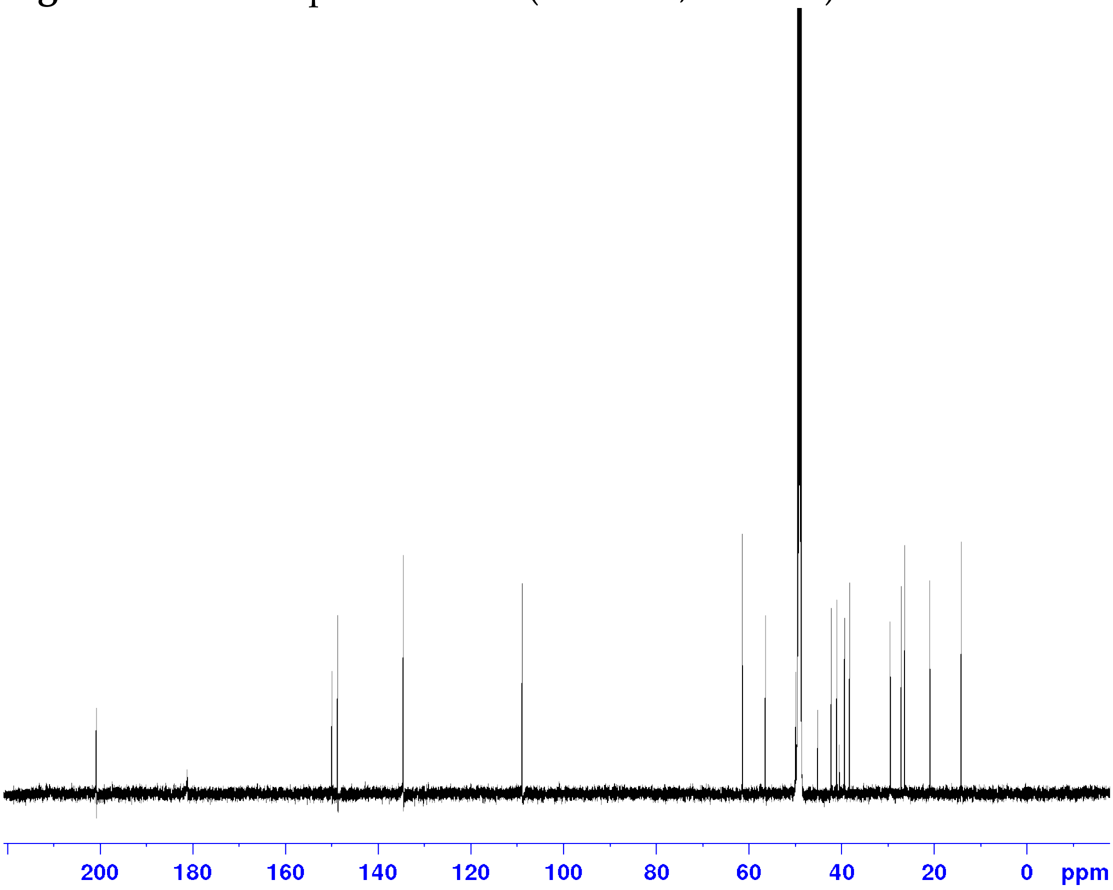

Fig. 3S. ^{13}C-NMR spectrum of **1** (150MHz, CD_3OD)

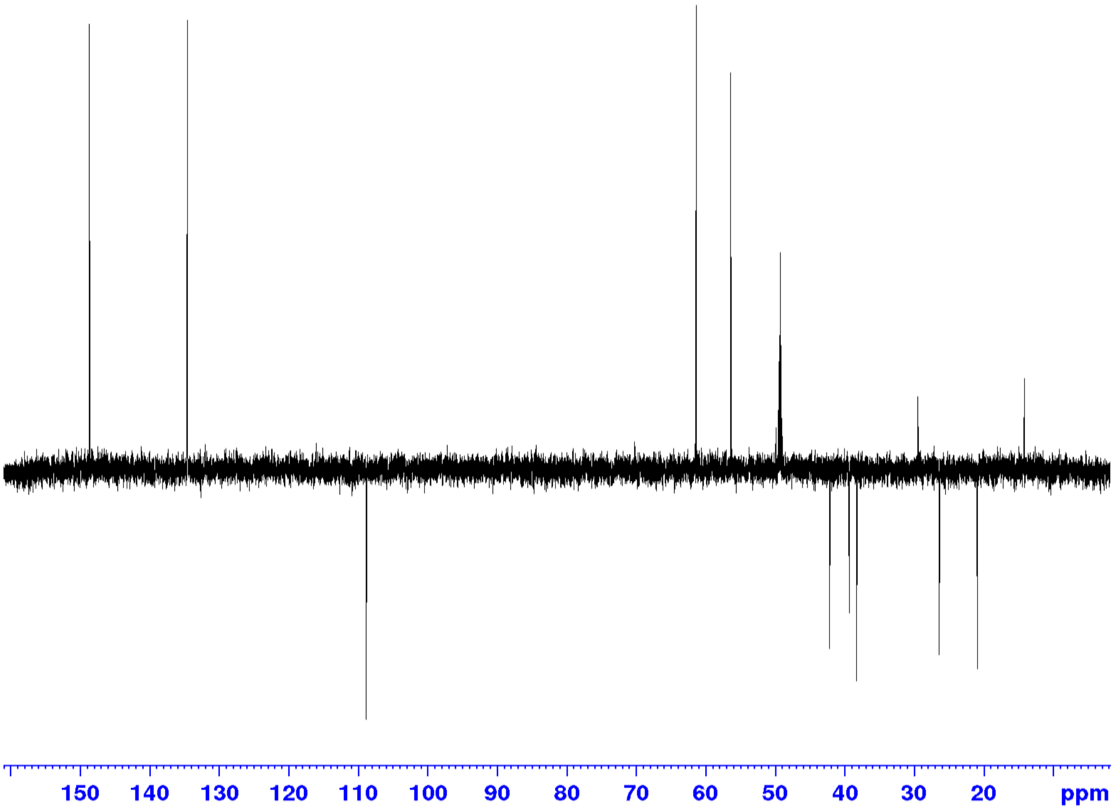

Fig. 4S. DEPT spectrum of **1** (150MHz, CD_3OD)

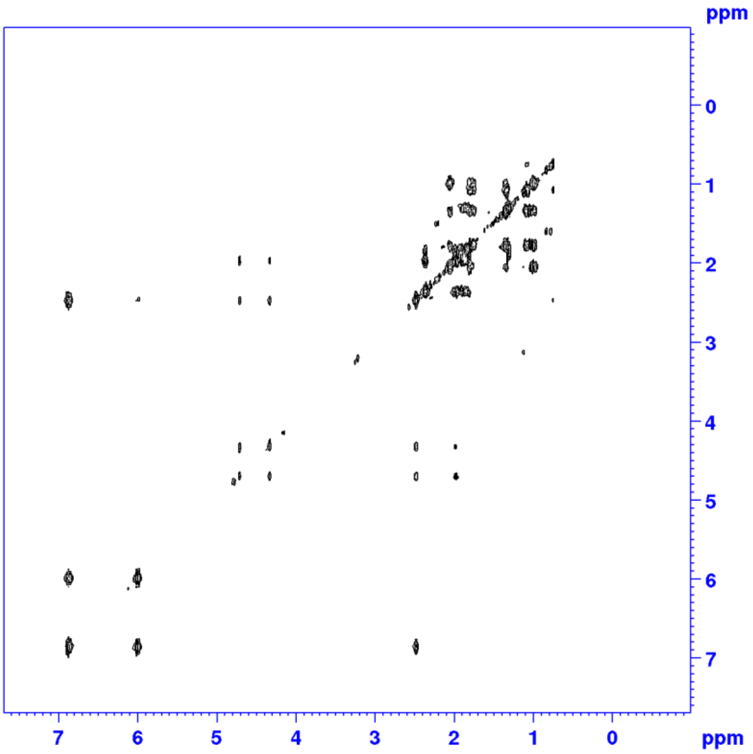

Fig. 5S. ^1H-^1H COSY spectrum of **1** (600MHz, CD_3OD)

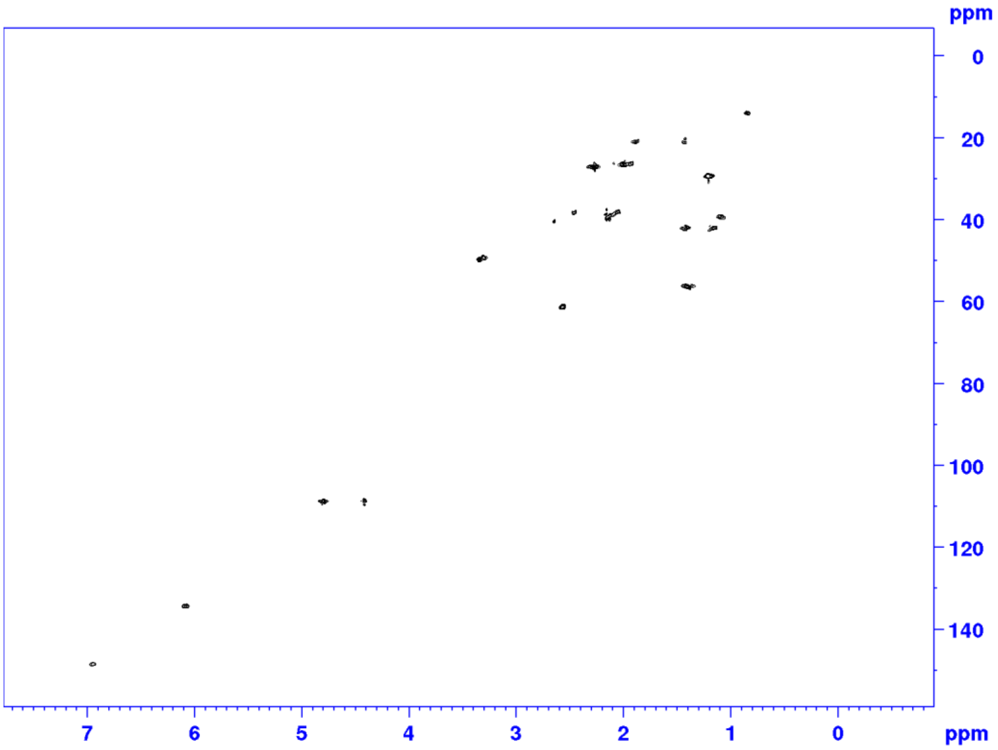

Fig. 6S. HSQC spectrum of **1** (600MHz, CD_3OD)

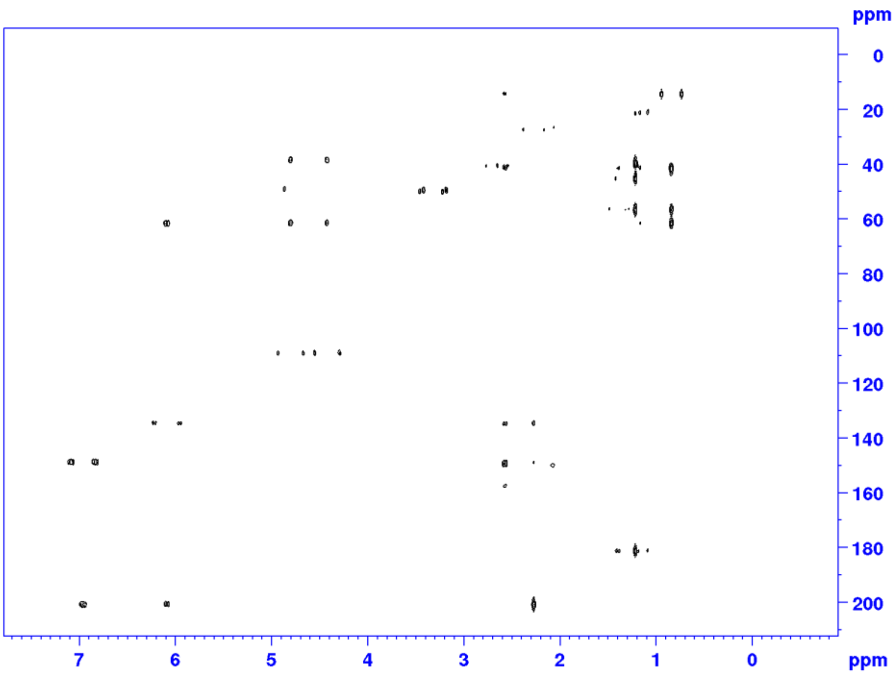

Fig. 7S. HMBC spectrum of **1** (600MHz, CD_3OD)

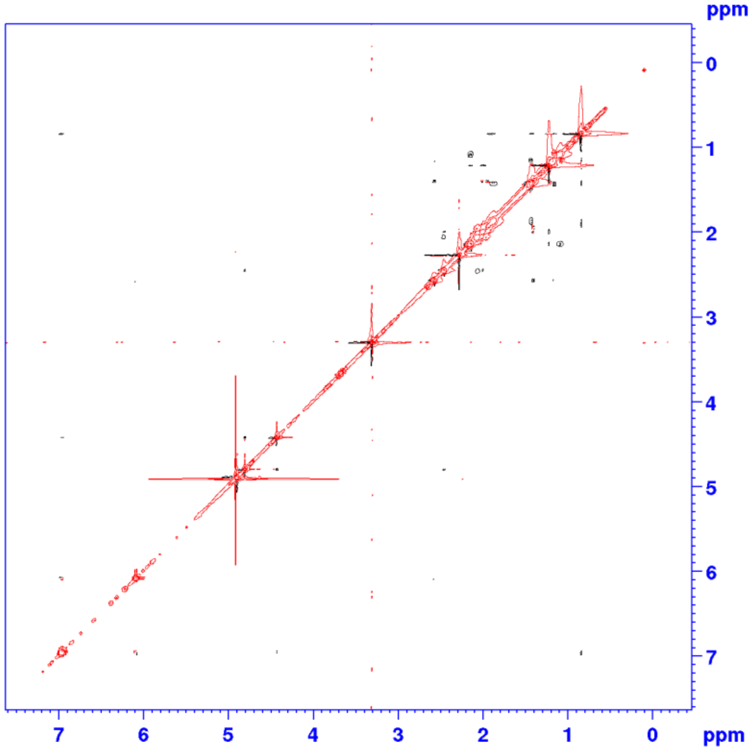

Fig. 8S. NOESY spectrum of **1** (600MHz, CD_3OD)

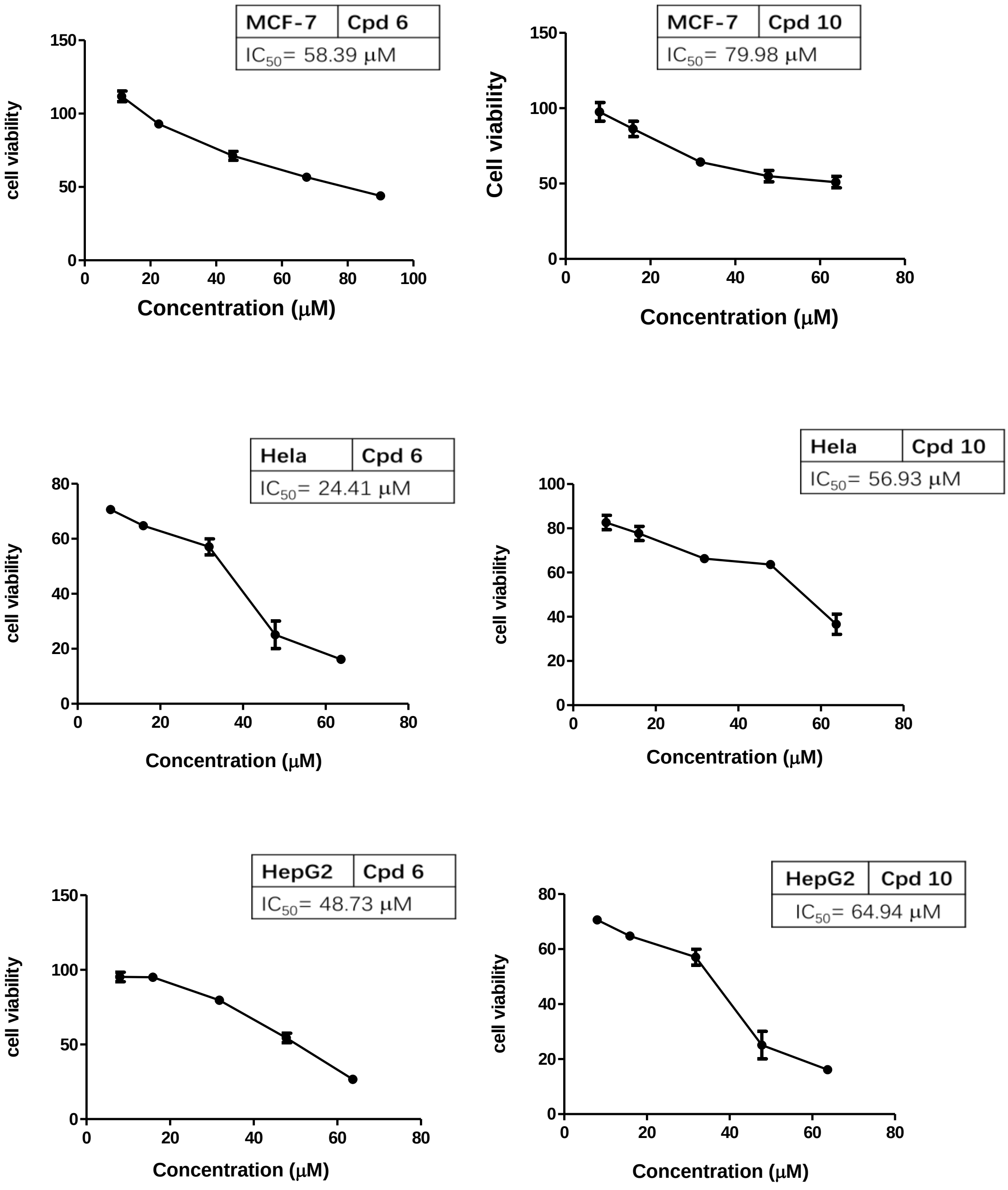

Fig. 9S. IC_{50} value determinations of active compounds

Table 1S. Crystal data and structure refinement for **3**

Empirical formula	$C_{20}H_{28}O_2$
Formula weight	300.42
Temperature/K	100.0(3)
Crystal system	orthorhombic
Space group	$P2_12_12_1$
a/Å	9.54890(10)
b/Å	12.6943(2)
c/Å	14.1587(2)
α/°	90
β/°	90
γ/°	90
Volume/Å^3	1716.27(4)
Z	4
ρcalcg/cm^3	1.163
μ/mm^{-1}	0.565
F(000)	656.0
Crystal size/mm^3	0.2 × 0.2 × 0.2
Radiation	CuKα (λ = 1.54184)
2 θ range for data collection/°9.356 to 148.94	
Index ranges	-11 ≤ h ≤ 11, -14 ≤ k ≤ 15, -15 ≤ l ≤ 17
Reflections collected	9031
Independent reflections	3362 [Rint = 0.0317, Rsigma = 0.0323]
Data/restraints/parameters	3362/0/205
Goodness-of-fit on F^2	1.054
Final R indexes [I>=2σ (I)]	R1 = 0.0331, wR2 = 0.0833
Final R indexes [all data]	R1 = 0.0371, wR2 = 0.0867
Largest diff. peak/hole / e Å^{-3} 0.22/-0.19	
Flack parameter	0.04(10)

Table 2S. Bond lengths for **3**

Atom	Atom	Length/Å	Atom	Atom	Length/Å
O1	C6	1.238(2)	C9	C10	1.557(2)
O2	C12	1.354(2)	C9	C1	1.540(2)
C11	C8	1.393(2)	C9	C20	1.543(2)
C11	C12	1.395(2)	C10	C5	1.527(2)
C14	C7	1.405(2)	C10	C4	1.560(2)
C14	C13	1.378(3)	C1	C2	1.530(2)
C8	C7	1.409(2)	C3	C4	1.536(3)
C8	C9	1.537(2)	C3	C2	1.520(3)
C7	C6	1.457(2)	C15	C16	1.532(3)
C6	C5	1.504(2)	C15	C17	1.520(3)
C12	C13	1.409(3)	C4	C19	1.541(3)
C13	C15	1.519(2)	C4	C18	1.537(3)

Table 3S. Bond Angles for **3**

Atom	Atom	Atom	Angle/°	Atom	Atom	Atom	Angle/°
C8	C11	C12	121.04(17)	C1	C9	C10	108.98(14)
C13	C14	C7	122.71(17)	C1	C9	C20	109.25(15)
C11	C8	C7	117.63(16)	C20	C9	C10	114.78(14)
C11	C8	C9	121.11(16)	C9	C10	C4	116.94(14)
C7	C8	C9	121.19(16)	C5	C10	C9	110.73(14)
C14	C7	C8	120.20(16)	C5	C10	C4	113.52(14)
C14	C7	C6	118.63(16)	C2	C1	C9	113.06(14)
C8	C7	C6	121.16(16)	C6	C5	C10	113.43(15)
O1	C6	C7	121.32(16)	C2	C3	C4	113.85(15)
O1	C6	C5	119.89(16)	C13	C15	C16	110.18(15)
C7	C6	C5	118.79(15)	C13	C15	C17	113.34(16)
O2	C12	C11	121.15(17)	C17	C15	C16	110.30(17)
O2	C12	C13	116.89(15)	C3	C4	C10	107.75(14)
C11	C12	C13	121.95(16)	C3	C4	C19	109.93(15)
C14	C13	C12	116.44(16)	C3	C4	C18	108.18(16)
C14	C13	C15	123.71(17)	C19	C4	C10	114.44(15)
C12	C13	C15	119.84(16)	C18	C4	C10	108.93(15)
C8	C9	C10	107.26(14)	C18	C4	C19	107.45(16)
C8	C9	C1	110.52(14)	C3	C2	C1	110.72(15)

C8	C9	C20	105.99(14)				

Table 4S. Crystal data and structure refinement for **6**

Empirical formula	$C_{20}H_{26}O_3$
Formula weight	314.41
Temperature/K	159.99(10)
Crystal system	orthorhombic
Space group	$P2_12_12_1$
a/Å	10.4261(4)
b/Å	14.6852(6)
c/Å	23.2228(12)
α/°	90
β/°	90
γ/°	90
Volume/Å^3	3555.6(3)
Z	8
ρcalcg/cm^3	1.175
μ/mm^{-1}	0.614
F(000)	1360.0
Crystal size/mm^3	0.2 × 0.15 × 0.15
Radiation	CuKα (λ = 1.54184)
2 θ range for data collection/°7.122 to 154.618	
Index ranges	-12 ≤ h ≤ 12, -10 ≤ k ≤ 17, -21 ≤ l ≤ 29
Reflections collected	12897
Independent reflections	6523 [Rint = 0.1032, Rsigma = 0.1098]
Data/restraints/parameters	6523/106/463
Goodness-of-fit on F^2	1.073
Final R indexes [I>=2σ (I)]	R1 = 0.0945, wR2 = 0.2573
Final R indexes [all data]	R1 = 0.1242, wR2 = 0.2949
Largest diff. peak/hole / e Å^{-3} 0.69/-0.45	
Flack parameter	0.2(3)

Table 5S. Bond lengths for **6**

**Atom**	**Atom**	**Length/Å**	**Atom**	**Atom**	**Length/Å**
O1	C5	1.380(8)	O4	C25	1.383(8)
O2	C6	1.254(7)	O5	C26	1.249(7)
O3	C12	1.354(8)	O6	C32	1.344(8)
C1	C2B	1.563(19)	C21	C22B	1.58(2)
C1	C9	1.547(8)	C21	C29	1.557(10)
C1	C2A	1.561(11)	C21	C22A	1.503(14)
C2B	C3	1.606(19)	C22B	C23B	1.52(2)
C3	C4	1.530(10)	C23B	C24	1.38(2)
C3	C2A	1.504(11)	C24	C30	1.524(9)
C4	C10	1.520(9)	C24	C38	1.646(12)
C4	C18B	1.56(2)	C24	C39	1.544(12)
C4	C19B	1.54(2)	C24	C38A	1.62(2)
C4	C19A	1.546(13)	C24	C23A	1.434(15)
C4	C18A	1.521(!3)	C25	C26	1.455(9)
C5	C6	1.461(9)	C25	C30	1.346(9)
C5	C10	1.346(9)	C26	C27	1.450(9)
C6	C7	1.453(9)	C27	C28	1.402(8)
C7	C8	1.407(8)	C27	C34	1.395(8)
C7	C14	1.402(9)	C28	C29	1.502(8)
C8	C9	1.518(8)	C28	C31	1.379(9)
C8	C11	1.386(9)	C29	C30	1.539(9)
C9	C10	1.514(9)	C29	C40	1.542(8)
C9	C20	1.559(9)	C31	C32	1.390(9)
C11	C12	1.376(9)	C32	C33	1.430(9)
C12	C13	1.434(8)	C33	C34	1.376(9)
C13	C14	1.370(9)	C33	C35	1.520(9)
C13	C15	1.497(9)	C35	C36	1.509(13)
C15	C16	1.584(15)	C35	C37	1.549(13)
C15	C17	1.489(13)	C35	C36A	1.79(3)
C15	C17A	1.59(4)	C35	C37A	1.46(3)
C15	C16A	1.52(4)	C22A	C23A	1.455(16)

Table 6S. Bond Angles for **6**

Atom	Atom	Atom	Angle/°	Atom	Atom	Atom	Angle/°
C9	C1	C2B	112.2(10)	C22A	C21	C29	117.0(9)
C9	C1	C2A	113.8(6)	C23B	C22B	C21	116(3)
C1	C2B	C3	108.1(14)	C24	C23B	C22B	100(3)
C4	C3	C2B	95.6(13)	C23B	C24	C30	112(2)
C2A	C3	C4	119.6(7)	C23B	C24	C38	101.8(19)
C3	C4	C18B	100(3)	C23B	C24	C39	125(2)
C3	C4	C19B	123(2)	C30	C24	C38	110.2(6)
C3	C4	C19A	102.2(8)	C30	C24	C39	110.2(6)
C10	C4	C3	112.5(6)	C30	C24	C38A	106.8(11)
C10	C4	C18B	104(4)	C39	C24	C38	94.2(7)
C10	C4	C19B	109(3)	C39	C24	C38A	134.3(14)
C10	C4	C19A	109.5(10)	C23A	C24	C30	114.1(9)
C10	C4	C18A	112.9(14)	C23A	C24	C39	106.3(7)
C19B	C4	C18B	107(2)	C23A	C24	C38A	81.4(14)
C18A	C4	C3	109.9(12)	O4	C25	C26	111.7(6)
C18A	C4	C19A	109.1(11)	C30	C25	O4	122.8(6)
O1	C5	C6	112.2(5)	C30	C25	C26	125.4(6)
C10	C5	O1	123.2(6)	O5	C26	C25	117.5(6)
C10	C5	C6	124.7(6)	O5	C26	C27	124.2(5)
O2	C6	C5	117.5(6)	C27	C26	C25	118.3(5)
O2	C6	C7	124.1(6)	C28	C27	C26	119.0(5)
C7	C6	C5	118.4(5)	C34	C27	C26	119.9(5)
C8	C7	C6	118.8(6)	C34	C27	C28	121.1(6)
C14	C7	C6	120.4(6)	C27	C28	C29	122.6(6)
C14	C7	C8	120.7(6)	C31	C28	C27	117.5(6)
C7	C8	C9	122.9(5)	C31	C28	C29	119.6(5)
C11	C8	C7	116.4(6)	C28	C29	C21	109.5(6)
C11	C8	C9	120.6(5)	C28	C29	C30	114.4(5)
C1	C9	C20	111.3(6)	C28	C29	C40	105.7(5)
C8	C9	C1	108.9(5)	C30	C29	C21	109.7(5)
C8	C9	C20	103.9(5)	C30	C29	C40	108.4(5)
C10	C9	C1	108.7(5)	C40	C29	C21	108.9(5)
C10	C9	C8	114.4(5)	C24	C30	C29	119.4(6)

C10	C9	C20	109.6(5)	C25	C30	C24	122.8(7)
C5	C10	C4	121.8(6)	C25	C30	C29	117.5(5)
C5	C10	C9	119.8(6)	C28	C31	C32	121.7(6)
C9	C10	C4	118.5(5)	O6	C32	C31	122.1(6)
C12	C11	C8	122.8(6)	O6	C32	C33	116.8(6)
O3	C12	C11	122.4(6)	C31	C32	C33	121.1(6)
O3	C12	C13	116.3(6)	C32	C33	C35	119.4(6)
C11	C12	C13	121.4(6)	C34	C33	C32	116.3(6)
C12	C13	C15	121.0(6)	C34	C33	C35	124.2(6)
C14	C13	C12	115.4(6)	C33	C34	C27	122.3(6)
C14	C13	C15	123.6(6)	C33	C35	C37	108.9(7)

Table 7S. The optimized conformation geometries, thermodynamic parameters, and proportions of **1**

No.1	**Conformation**	**Proportion**
1	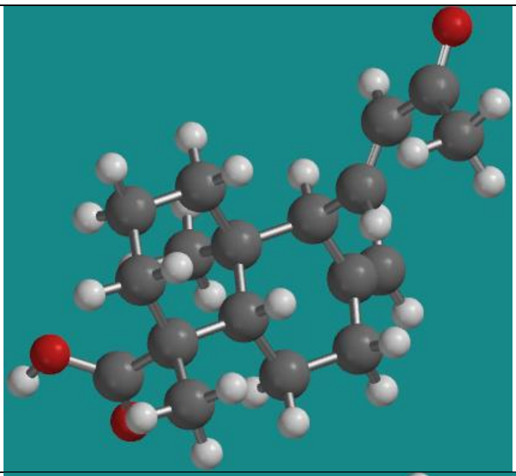	52.5%
2	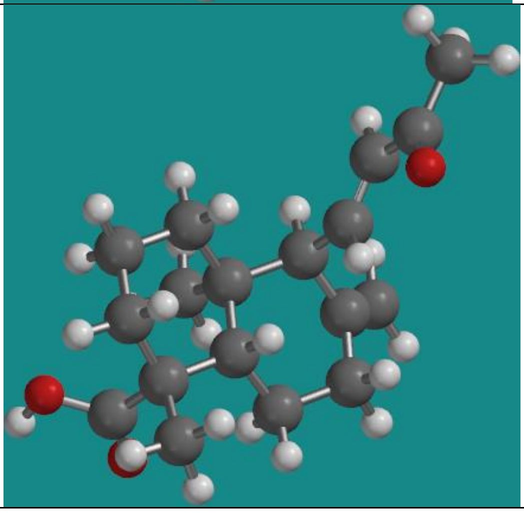	47.5%

3	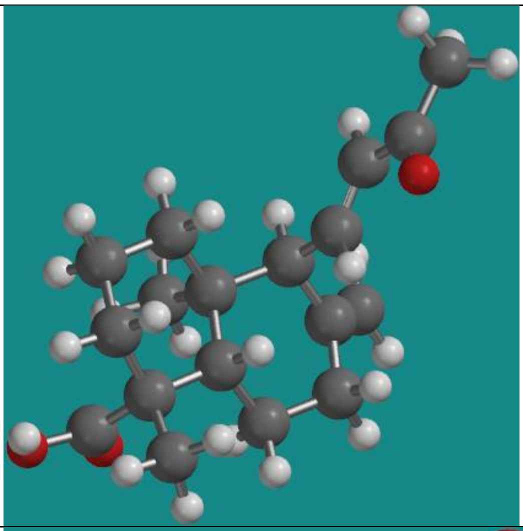	0%
4	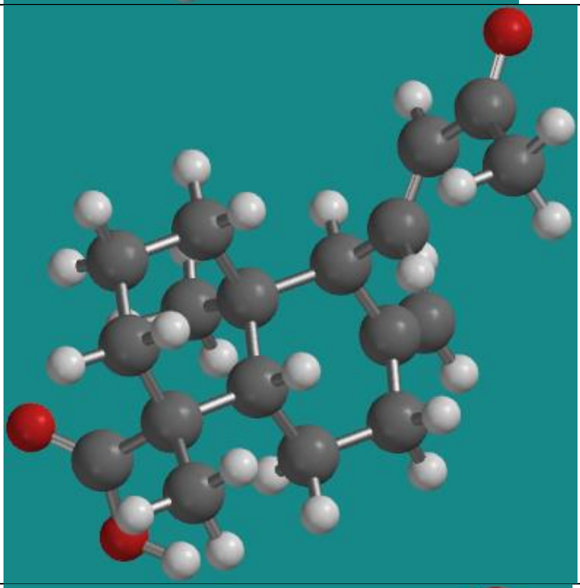	0%
5	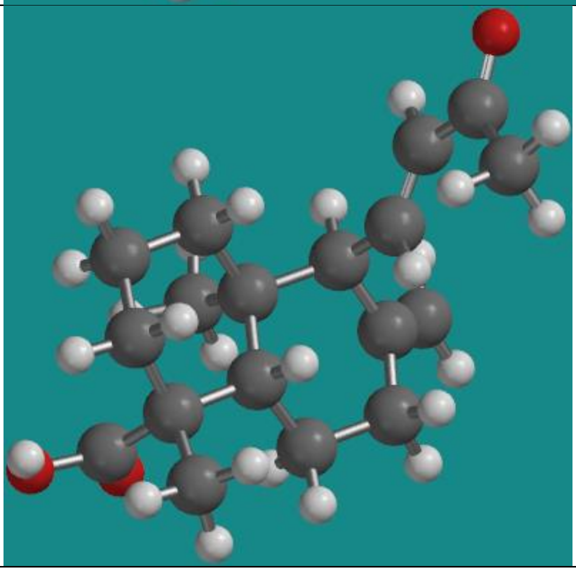	0%
